# Health Equity in Pediatric Drug Development: Translating Aspiration into Operation

**DOI:** 10.1007/s43441-022-00410-3

**Published:** 2022-05-20

**Authors:** Morenike Oluwatoyin Folayan, Magda Conway, Carolyn Russo, Nilza Diniz, Lungile P. Jafta, Nadia A. Sam-Agudu, Sarah Bernays, Victor M. Santana, Carla Epps, Mark A. Turner

**Affiliations:** 1 Department of Child Dental Health, Obafemi Awolowo University, Ile-Ife, Nigeria; 2 PENTA, Corso Stati Uniti 4, 35127 Padua, Italy; 3 Department of Hematology, St. Jude Children’s Research Hospital and Comprehensive Cancer Center, Memphis, TN USA; 4 Biology Department, Biological Sciences Center, State University of Londrina, Londrina, PR 86057-970 Brazil; 5 International Research Center of Excellence, Institute of Human Virology Nigeria, Abuja, Nigeria; 6 Institute of Human Virology and Department of Pediatrics, University of Maryland School of Medicine, Baltimore, MD USA; 7 Department of Pediatrics and Child Health, University of Cape Coast School of Medical Sciences, Cape Coast, Ghana; 8 School of Public Health, University of Sydney, Sydney, Australia; 9 Department of Global Health and Development, London School of Hygiene and Tropical Medicine, London, UK; 10 Departments of Oncology and Global Pediatric Medicine, St. Jude Children’s Research Hospital and Comprehensive Cancer Center, Memphis, TN USA; 11 Office of Pediatric Therapeutics, Food and Drug Administration, Silver Spring, MD USA; 12 Institute of Life Course and Medical Sciences, University of Liverpool, Liverpool Health Partners, Liverpool, UK; 13 Neonatal Unit, Liverpool Women’s NHS Foundation Trust, Crown Street, Liverpool, L8 7SS UK

**Keywords:** Child, Adolescent, Drug development, Stakeholders, Engagement, Health equity, Access

## Abstract

The concept of health equity—the attainment of the highest possible level of health for all members of society—requires equitable access to all aspects of healthcare, including pediatric drug development. However, many communities are under-represented in pediatric drug development programs. Barriers to participation include geographic, economic, racial/ethnic bias, legal, cultural, linguistic, and other factors. While there is no “one size fits all” approach to addressing these barriers, community engagement and collaboration is recognized by the Centers for Disease Control, the World Health Organization, and other global health organizations as a cornerstone for building a more equitable healthcare system. In this article, we will present case studies of stakeholder and community engagement in clinical research for rare diseases and other areas of healthcare, as examples of strategies and practices for actively involving under-represented communities and fostering their participation in pediatric drug development programs. These studies may serve as templates for facilitating equity in pediatric drug development from aspiration into operation.

## Introduction

Medicines are developed in order to benefit individuals and society at large. The issues of diversity and equity in patient participation are increasingly receiving attention from stakeholders in drug development. According to Pharmaceutical Research and Manufacturers of America (PhRMA), “The biopharmaceutical industry is committed to driving real change and improving health outcomes for patients in underserved communities” [1]. Regulatory authorities are also developing regulatory strategies for promoting inclusion of under-represented populations in clinical trials. For example, the Food and Drug Administration (FDA) has published a guideline on “Enhancing diversity in clinical trial populations” [2]. Other stakeholders have identified the need for inclusive approaches to research [3, 4]. However, there is limited experience of practices/strategies relating to engaging diverse and underserved communities, particularly during pediatric drug development. The purpose of this paper is to promote the inclusion of underserved and historically excluded individuals and communities in the design and conduct of pediatric clinical trials by sharing some relevant experiences. Accordingly, its objectives are as follows:

1. Present an overview of justice and equity as drivers for the inclusion of underserved communities
2. Share three case studies that describe efforts to address multiple barriers to geographical, legal, and cultural diversity in the equitable representation of children and adolescents in pediatric drug research.
3. Outline lessons learned and make recommendations based on the case studies.

### Justice, Equality, and Equity in Pediatric Drug Development

Justice, equality, and equity are core principles for human societies. The universality of these principles is codified in the Universal Human Rights Declaration (United Nations 1948) [5], which outlines the rights and freedoms of individuals in a just society, and the Universal Declaration on Bioethics and Human Rights, which aims “to promote equitable access to medical, scientific and technological developments [6].”

Philosophers have proposed several theories of justice [7]. For example, Aristotle regarded justice as a fundamental virtue for a society that is free from conflict and is ordered and predictable. Embedded in justice are several concepts such as legal justice, particular justice, and corrective justice. Some of these concepts point towards equality, where everyone gets the same resource (e.g., a fixed stipend amount for all scholarship recipients). However, equality is not enough to meet all the concepts of justice, which also includes the notion of fairness. All human populations have some degree of variability such as in genetic constitution, social and economic status, culture, and religion. Thus, equal treatment does not lead to equal access to good health. Rather, we need to consider equity as well as equality. Whitehead describes equity as the situation in which “…ideally everyone should have a fair opportunity to attain their full health potential and, more pragmatically, that none should be disadvantaged from achieving this potential, if it can be avoided [8].” Equity is therefore concerned with creating equal opportunities for health, and with bringing health differentials down to the lowest level possible [8].”

Health equity for populations requires access to research, and particularly access to results of research that are applicable to each population. Many populations do not have access to relevant research findings because funding [9], study design, recruitment, retention, analysis, and interpretation together with implementation of results and technology transfer after successful research are not done equitably [10]. In many parts of the world current or historic inappropriate practice, including overt or hidden racism, underpins inequity in healthcare and research. Researchers may select some regions for research because of reduced safeguards for study participants; this approach reduces willingness to participate in research particularly when the benefits of research are not shared equitably [11]. Inappropriate practice is remembered within communities and often affects choices that communities and individuals make about research [12–15].

Therefore, pediatric drug development strategies should account for justice and equity in access to clinical trials for individuals from all segments of society. These considerations underlie the pursuit of equity in pediatric drug development in the three case studies.

## Methods

Case studies were selected through informal contacts made by the senior authors (CE and MAT). The case study descriptions were elaborated iteratively through video conferences between all the authors and within the drafting group for each case. The descriptions were informed by the Patient Engagement Quality Guidance (PEQG) tool [16]. The PEQG tool was developed by the Patient-Focused Medicines Development initiative, a not-for-profit collaborative organization to benefit patients and health stakeholders by encouraging patient-centered healthcare systems, [17] but this format was not followed completely. The lessons learned were identified by the senior authors.

## Case Studies

### Case Study 1

#### Overcoming Geographical Inequity: The St. Jude Hospital Affiliate Program in Pediatric Oncology Research, USA

##### Background

Every pediatric cancer is a rare disease [18]. Treating rare diseases with curative intent requires systematic investigation which is made possible through the conduct of clinical trials. Clinical trials reduce haphazard, non-standard treatment practice that expose children to potential harm and are unlikely to advance cures [19]. Enrollment in clinical trials should include participants that represent the diversity of the population affected by the disease, since the results must be generalizable to the population at risk.

In contrast to adult oncology, most pediatric oncology clinical trials in the United States are conducted within a framework that includes a partnership between clinical trial networks such as National Cancer Institute cooperative groups, government, academic centers, and industry. Most pediatric cancer clinical research trials are conducted in academic medical centers located in major urban centers, and thus, often recruit study participants proximal to their location of practice. This recruitment practice may be subject to bias in geographical representativeness. It introduces bias into the clinical trial, as study cohorts may not represent the diversity of the ‘real-world’ circumstances and patient experience. Thus, an important clinical trial enrollment barrier is access limited by geography [20, 21]. In the United States, 20% of the population resides in rural areas. However, only 9% of physicians practice in these settings. The percentage drops to 3% for oncology specialists and drops further for pediatric oncologists [22]. Children living in rural America do not have the same access to clinical trials as children in urban centers [23] and racial and ethnic minorities comprise 22% of rural populations in the United States [24]. Despite efforts to increase access to clinical trials, rates of participation by socio-economically disadvantaged and historically excluded groups remain low [24].

##### Activity Description

St. Jude Children’s Research Hospital (St. Jude), Memphis, Tennessee, US, advanced an approach of well-designed clinical trials starting in the 1960s when the cure rate for acute lymphoblastic leukemia was 10%. Today, childhood acute lymphoblastic leukemia is a curable disease, with greater than 95% of children surviving their disease [25]; the Affiliate Program at St. Jude has contributed significantly to this process. The goal of the Affiliate Program at St. Jude is to facilitate equal access to pediatric oncology care regardless of geographic location. Our research in health disparities has shown that the affiliate model benefits all children at the sites. We found that there is no difference in event-free survival for black children as compared to white children with acute lymphoblastic leukemia after adjusting for high-risk features [26]. Providing equity in healthcare mitigates this risk and improves survival for children.

The Affiliate Program is driven by the St. Jude vision that no child is denied treatment based on race, religion, or a family’s ability to pay. St. Jude aims to advance science in the context of clinical need and the alignment of research and care underpins the actions of the Affiliate Program. Established in 1999, the Affiliate Program allows more children equal access to pediatric oncology care through clinical trial protocols in a closer-to-home community setting located throughout the Southeast and the Midwest United States. These clinics serve diverse patient populations (including Hispanic, American Indian, African American, and socially disadvantaged patients) in rural and suburban communities. The clinic reach is approximately 350 patients per year, accounting for nearly 40% of the clinical trial enrollment at St. Jude. The Affiliate Program has helped to mitigate barriers to clinical trial enrollment imposed by long-distance travel, which could otherwise have been a burden to working parents or parents of multiple children. Time away from work for parents is difficult, even with the benefit of the Family Medical Leave Act. Furthermore, time away from other children and lack of social support affects quality of life for the family unit.

##### Methods

The journey to the current program started before 1999 with informal personal contacts with physicians attending educational opportunities at St. Jude. The program evolved over many years driven in the parallel but integrated tracks of clinical care and research. The Affiliate Program takes an integrated approach to facilitate cancer care for patients and families. This model is supported by other key components that include patient navigators who assist with care collaboration and the patient transition. Similarly, a family advisory committee that includes parents from the affiliate communities provides feedback to continually improve the process for family and caregivers. Leveraging digital health tools also provides an opportunity to lessen travel burdens for trial participants, particularly for those living in rural areas with limited access to major cancer centers.

When reaching out to prospective partners (healthcare systems or hospitals) for the affiliate program, one of St. Jude’s primary considerations was the partners’ commitment to their community and shared commitment to clinical research. They shared with St. Jude a mutual professional interest in leveraging existing systems in their community to establish the necessary infrastructure for community-based oncology care. These partners recognized that there was a strong geographical need for pediatric oncology services and that there were gaps in the existing infrastructure (e.g., no pediatric oncologist) in their community.

Once a firm commitment was made with a community hospital partner, St. Jude performed a formal site assessment to identify gaps and needs with respect to supportive systems (processes, workstreams, efficiencies, and community support), education and training about research in the partner, and infrastructure for clinical research. Resources are allocated based on the identified needs, sites are reviewed at regular intervals, and the partnership is formalized through legal review and execution of contracts. The sites formally open for operation in the community about 18–24 months after the start of the formative assessment. The first affiliation was formalized in 1999.

The network has been sustained by a system that effectively shares the successes of the outcomes of improved pediatric oncology care through effective community. Communication with partners is done on a scheduled arrangement (weekly, monthly, quarterly, and annually dependent on the activity and opportunity) using e-mail, webinars, and in-person events. Affiliated staff also have the opportunity to receive quality improvement support and training. This collaborative approach maintains buy-in through self-directed improvement and ownership of projects by affiliates.

### Case Study 2

#### Overcoming Legally-Based Inequity in Research Participation for Fostered Minor Children: Developing Inclusive Ethics Guidelines in West Africa

##### Background

It is not uncommon for African children to live with non-parental adults in informal fostering or kinship care settings [27]. In West Africa, between 11 and 29% of children are raised by fostering adults who help to reduce financial and other caregiving burdens of biological parents and/or relatives [28]. Some children—such as Almajiris or Talibѐs—live under the care of non-parental adults for religious reasons [29, 30]. The HIV/AIDS epidemic has also contributed to higher rates of child fostering. In 2019, an estimated 3.27 million under-18 children in West and Central Africa lost one or both parents to HIV/AIDS [31].

The participation of African children in clinical trials for drug development—including for HIV—has been sub-optimal [32]. This is partly due to ethical consent challenges for under-18 children [33–35], especially for adolescent participation in HIV and sexual and reproductive health research [33, 36–38]. Research ethics principles consider minors incapable of autonomous decision-making, thus researchers are to seek parental permission as consent-by-proxy for minors to participate in research [39, 40]. When biological parents are unavailable, legally-authorized representatives provide parental permission in lieu of the former [41].

In the United States, a legally-authorized representative in the context of pediatric research is “an individual, or judicial or other body authorized under applicable law” to grant consent-by-proxy for a prospective participant below the legal age of majority, when their biological parents are not available [41]. If there is no applicable law governing legally-authorized representatives, an individual considered acceptable by “institutional policy” can provide parental permission for the minor [41]. In West Africa, adult caregivers fostering minors can be considered, and often serve as legally-authorized representatives, albeit typically outside the law. When research teams/institutions do not recognize fostering legally-authorized representatives as suitable to give parental permission, minors may be unduly excluded from studies. The principle of justice in biomedical research supports the inclusion of all populations that would potentially benefit from a study [40]. In the absence of operational considerations for children without access to legally-authorized representatives (as defined by applicable laws), there is the risk of excluding from research significant proportions of often highly vulnerable children in West Africa who live with non-parental adults. Locally relevant guidance for fostering and non-fostering legally-authorized representatives is needed to guide research ethics committees in West Africa.

##### Activity Description

In September 2017, twenty-four members of the Network of Ethics Committees Operating in West Africa (NECOWA) met to discuss ethical considerations for research protocol review during humanitarian emergencies. One of the critical issues raised was the recognition and identification of legally-authorized representatives for minors. NECOWA is developing a legally-authorized representatives consensus working document to present to the West Africa Task Force on Emergency Responses and the West African Health Organization for further deliberation by national, state/provincial, and institutional ethics committees in West Africa. Country-level adaptation of NECOWA’s recommendations are to be integrated into national bioethics policies. A follow-up survey among NECOWA members and selected institutions will assess the application of the legally-authorized representatives consensus guidelines in ethics committee policies and review.

The process to develop the consensus document started shortly after the 2017 meeting and was led by the New HIV Vaccine and Microbicide Advocacy Society. An iterative consultative research process was instituted, with the objectives to (1) reach a consensus on an operational definition of legally-authorized representative suitable for research in West Africa; and (2) define the process of institutionalizing the operational definition of legally-authorized representative by research regulatory agencies in West Africa. The Delphi consultation process was adapted to facilitate an expert-led process to achieve these objectives [42], and a community participatory approach will also be used to validate the outcomes of the iterative process.

##### Methods

Eligible expert Delphi participants were bioethicists and pediatric/adolescent health researchers working in Africa, particularly West Africa, who had published ≥ 5 papers. To identify them, we conducted a systematic search of Pubmed, Google Scholar, and African Journals Online for bioethics and pediatric/adolescent health literature published between 2000 and 2018. Other experts were recruited from editorial boards of bioethics-focused journals. Ethics committee members and policymakers involved in international, regional, and national decision-making on pediatric/adolescent health were also identified as critical stakeholders for this process. Finally, NECOWA members and pediatric/adolescent health policymakers identified through 2010 to 2018 WHO policy document review were also recruited. The final Delphi participant group will be determined through a process of selection for equitable representation of gender, research field, Anglophone, Francophone, Lusophone West African representation, level of research expertise, and community advocacy/community-based research experience. To date, this process has been self-supported exclusively by the authors.

To complete the process, data will be generated through five study phases conducted over 32 weeks. *Phase 1* (4 weeks) will involve a virtual meeting of 25 pediatric/adolescent health researchers, bioethicists, social scientists, and civil society representatives working with children and adolescents in West Africa. They will help clarify themes to be explored for the study, develop research questions, and fine-tune study methodology. In *Phase 2* (10 weeks), core research questions identified in Phase 1 will be developed into a quantitative survey tool to explore public perceptions of the questions. The tool will take the form of an online survey that will be open to the global public for 6 weeks. This survey will also provide insight on how engagement of the larger public community can enhance ethics committee decision-making regarding legally-authorized representatives. *Phase 3* (4 weeks) will host a second virtual discussion by the Phase 1 panel, covering findings from the survey, with the aim of reaching consensus on the concept of legally-authorized representative appropriate for the West African context. A draft 1.0 consensus document will be shared with 30–35 experts working in the field of epidemiology or clinical research with children and adolescents in West Africa, for their comments. A draft 2.0 consensus document will be developed; and in *Phase 4* (4 weeks), it will be shared with 20–25 experts who develop national, regional, and international policies on pediatric and adolescent health research for their review and feedback. The resulting draft 3.0 document will be shared with approximately 60 identified community stakeholders and the public (using social media platforms) during *Phase 5* (10 weeks). Comments received from the stakeholders and public will be used to develop the final iteration of the consensus document for publication and dissemination.

### Case Study 3

#### Overcoming Lack of Equitable Influence by Young People on Research: Changing the Culture of Clinical Trials in the United Kingdom, South Africa, Uganda, and Zimbabwe

##### Background

Young people have been poorly engaged in the design and development of interventions aimed at helping them [43]. Limiting their influence dismisses their knowledge of their own experiences and their understanding of what initiatives will likely be effective for them. Youth engagement aims to increase the influence of youth on the design, delivery, and implementation of youth-focused interventions, including clinical trials. Youth engagement in the design and implementation of youth-related health research, including clinical trials and programs is gaining attention in increasing number of countries and settings worldwide [44]. However, efforts at engaging young people in the design and implementation of clinical trials had been ‘tokenistic’ [45, 46], with institutions having created little or no capacity building for research-related youth engagement processes till date. Movement from a tokenistic to a fully realized model of youth engagement in clinical trials research requires investments towards building the skills and competency of representatives of young people in these spaces so that they are able to participate in a meaningful and effective manner.

While monetary concerns can be a consideration in developing youth engagement processes, there are also significant cultural barriers to making this paradigm shift. There is also potential anxiety within the research community about engaging children and young people in governance due to concerns about how well they could understand scientific details. Researchers may also be concerned that if children and young people were allowed to influence research decisions, what they might say might conflict with adult researchers [47, 48], which may in turn disrupt conventional practice.

This case study looks at young people living with HIV, their engagement in the development and delivery of pediatric HIV clinical trials in four countries, and the cultural changes that need to happen across the clinical trials enterprise if young people are to influence research.

##### Activity Description

**Fig. 1:**
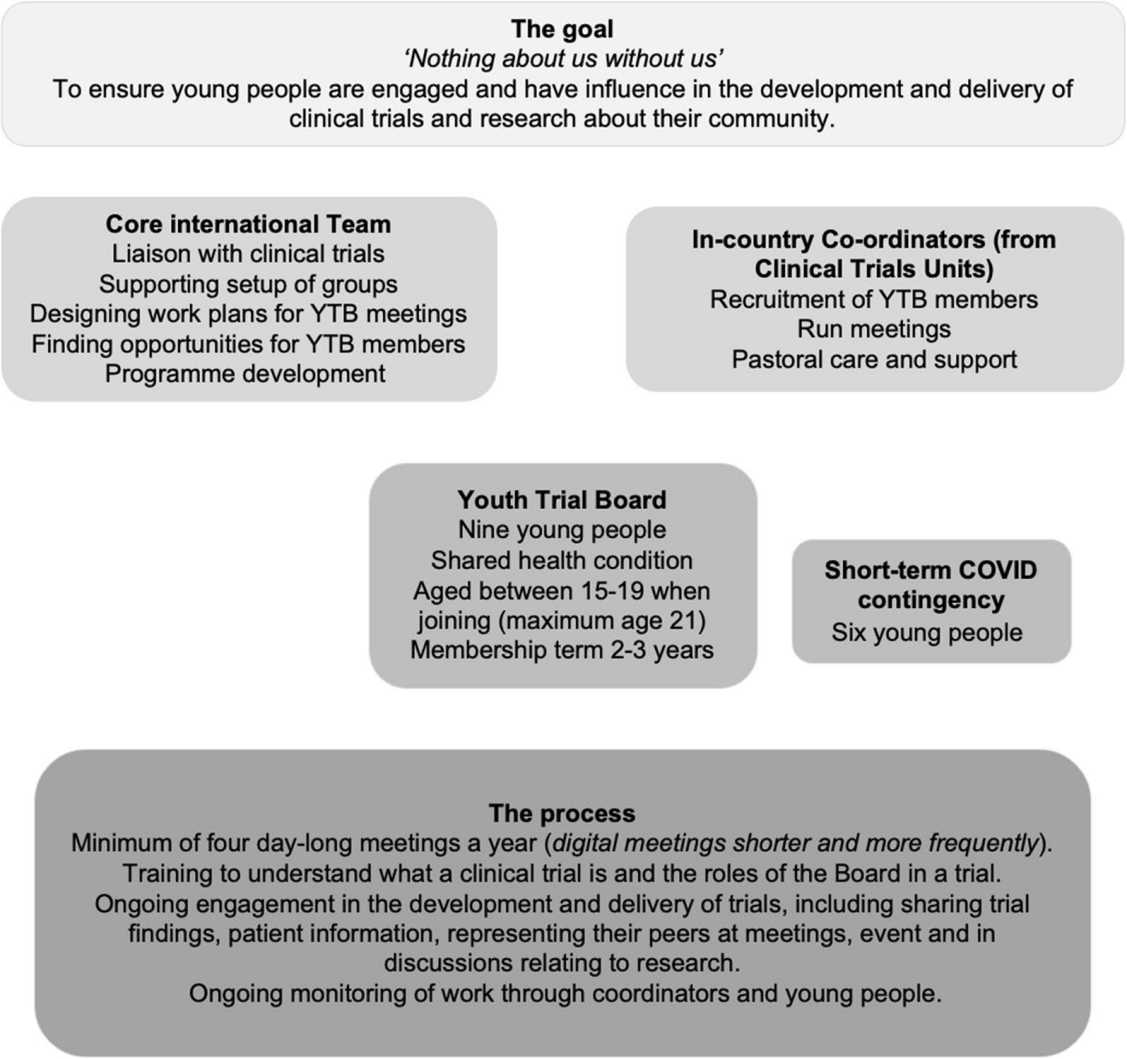
Youth Trial Boards and culture change during pediatric drug development

The goal is to increase the influence of young people in clinical trials that are about them and their community. Providing skills training to young people who represent their communities in clinical trial management systems will have multiple impacts. The Youth Trials Board (also known as YTBs) is an example of a group of young people who improved youth-focused HIV treatment research. Youth Trials Boards are groups of young people between 15 and 19 years old who are trained to engage in the development of pediatric HIV clinical trials to ensure that they reflect and respond to the needs of young people themselves (Fig. 1). They undertake many of the same tasks as Young People’s Advisory Groups, YPAGs, (see Preston et al. this issue—editors to provide reference information, DOI, etc.) but draw on both their lived experience of a specific condition and their ongoing engagement with healthcare as part of the management of chronic conditions that affect their peers. Youth Trials Board members are selected to ensure that Youth Trials Boards represent the diversity of adolescents, with specific attention paid to including those typically under-represented. Youth Trials Boards are intended to be a mechanism through which young people can engage with clinical researchers and meaningfully influence research concerning them.

##### Methods

The Youth Trials Board project began in 2014 and is ongoing. The initial step of the project—securing industry funding—took two years because of funders’ hesitancy since this was a novel approach. The next step of the project was to develop an explicit model and implement a pilot program. A literature review and formative research with young people informed the foundational theory which underpinned the development of the preliminary model for Youth Trials Boards. This was further informed by close collaboration with clinical trial teams who provided valuable input. The Youth Trials Board model was piloted in four countries—the United Kingdom, the United Kingdom, South Africa, Uganda, and Zimbabwe—over a period of three years through a sub-study of ODYSSEY, a clinical trial evaluating dolutegravir-based antiretroviral therapy in HIV-infected pediatric patients [49]. Because the project was multi-national, a critical step in launching the pilot was the development of joint practice agreements with project partners that met country-specific regulations and laws. Initially, two groups were based at Clinical Trials Units (CTUs) and the other two at non-governmental organizations. During this stage of implementation, we observed that having a group embedded in a CTU better facilitated project processes and communication; therefore, all the groups were moved into CTUs.

Each Youth Trials Board had two coordinators from the CTU who strived to ensure the young people participating were representative of the demographics of pediatric HIV infection in each country. The boards are actively engaging young people from low socio-economic backgrounds. Over the first year, each of the four groups participated in a series of modular trainings. A social scientist joined these sessions to observe and take field notes, running reflection sessions during the training days to collate the views and ideas of young people. These reflections were iteratively fed back to the Project Coordinator to inform and revise the design of the Youth Trials Board model. The Youth Trials Board training course was co-created through this process with the input of 36 young people living with HIV from the four countries.

Once established, the groups held four meetings a year at which they undertook various activities relating to research. The pilot focused on training, preparing, and supporting young people to engage as equitable partners in discussions about clinical trials and research. This preparation helped to partly overcome the conventional and intimidating power hierarchy that young people experienced during these formal events. YTBs were able to voice their opinions and at times influenced the practice and approach of clinical researchers. An example is the Youth Trials Board review of the 11-page Patient Information Sheet (PIS) developed for the trial sites. The groups noted that the sheet needed to be concise, understandable, and user-friendly to ensure genuinely informed consent. One Youth Trials Board member provided the following written feedback: ‘*feel treated like I’m stupid, because they know I can’t read it all.*’ The outcome of the PIS review was that Youth Trials Board groups created infographic information to replace the document. This activity is an example of an instance where the pilot worked, but overall the access and influence available to the young people involved was limited, despite the fact that they were trained, willing, and highly capable.

Additional funding was sourced to continue the work and focus on the cultural changes needed in clinical trials. A core team was established staffed by individuals with a passion for working with youth, embedding the youth participation ethos at the center of the work. These individuals come from various backgrounds and disciplines, including individuals with knowledge and understanding of children’s rights, youth-work models, and youth participation; the core team also includes a young person living with HIV. The role of the core team is to oversee all Youth Trials Boards, work closely with international trials and research, progressing the model and supporting the onsite coordinators to work with the young people. Each site has two coordinators recruited from existing CTU staff. The coordinators administer the Youth Trials Boards, run the meetings, and provide members with support. Support is needed because a key component for any work with young people who face adversity and stigma due to their health condition is ensuring their overall well-being, including their mental and emotional health. This was partly lost from moving the groups from the NGOs, which operated within a health and wellness model to CTUs, which operated within a medical model. To ensure that overall health and wellness are not overlooked, the CTU site contracts include the need to establish a referral system for Youth Trials Board members who may need support with health and wellbeing. Each CTU had a list of services/providers available in the community but no formal contract about service provision and/or funds to cover these services. Future work needs to address these gaps. In addition, Youth Trials Board meeting plans now start with an emotional ‘check-in’ activity.

COVID-19 led to the development of a digital model of engagement wherein Youth Trials Board members were provided access to digital equipment, data, and trained on the use of technology. Engagement of Youth Trials Board continued through a hybrid of digital and face-to-face meetings. Face-to-face meetings were held only where country COVID-19 control policy allowed for in-person contact. Program work is now focused on engaging clinical trials teams to understand how young people’s influence can be improved and increased. Specifically, the work will explore the format, structure, and decision-making process of trials. For example, the project is addressing the role of young people in clinical trial governance by working with four trials to develop a structure and support package so that older young people can be members of Trial Steering Committees (who are autonomous groups whose role is to provide overall supervision for a trial on behalf of the Trial Sponsor and Trial Funder) [50]. In 2022, the project will publish ‘Quality Standards on Youth Engagement in Clinical trials and cohort studies (working title),’ focusing on funders, strategists, and those running clinical trials. In addition to the ODYSSEY trial, the program is now working closely with other pediatric HIV trials (BREATHER+  [51], D3 [52], SHIELD [53], and cohort study REACH [54]).

To date, the most fruitful impact of this work has been raising awareness and initiating conversations with all key stakeholders on the value and benefits of engaging with and listening to the opinions of young people. Specifically, the project has highlighted how this can strengthen trial structures and mechanisms, and the necessity of engaging young people at all points of this process in order to be able to do that effectively. Examples of this are the PIS review activity, which led to the development of youth-led information using imagery, cartoons and infographics, supporting young people to understand complicated information. The PIS review activity demonstrated to other key stakeholders how ‘business as usual,’ where we produce the equivalent to adult-based resources and just adapt the language, may be insufficient to adequately engage and communicate with young people.

The impact of the project was assessed through embedding social scientists at each site that attended all the Youth Trials Board meetings in the pilot stage and conducted interviews with Youth Trials Board members and facilitators at the end of the pilot. Through these assessments, we were able to demonstrate the emerging value of the pilot—where young people felt it was really important to be part of the process and that this was a good mechanism to recognize their opinions and experiences by other key stakeholders in the process. These assessments helped us begin to understand what the process was achieving as well as what additional data needed to be collected in order to conduct a comprehensive and robust evaluation of the program. The program is now collecting additional data, including tracking young people’s experiences.

A key challenge that emerged during the pilot evaluation was the unmet expectations of the young people and the need to manage expectations of how quickly Sponsors can change their approach to trials. The pilot taught us that there was a great deal of initial enthusiasm and optimism, but also a risk that young people may be disappointed due to the pace of change (i.e., that the impact may not be immediate or explicit). Managing this expectation and ensuring that young people understand the process is now an essential element of the training.

## Discussion: Lessons Learned

The case studies provide evidence on how geographical residential location, legal status, and age can be a barrier to children and adolescents recruitment and engagement with the design and implementation of pediatric drug clinical trials as summarized in Table 1.

**Table 1:** Developing successful community engagement programs: lessons learned

Lesson learned	Case study	Examples
Address a clear need shared by multiple stakeholders	Saint Jude Affiliate Program, USA	Need to improve outcomes of cancer treatment irrespective of location
	Legally-authorized representatives, West Africa	Need to recognize and identify legally-authorized representatives of minors for research conducted during humanitarian emergencies
	Youth Trial Boards (United Kingdom, South Africa, Uganda, Zimbabwe)	Need to engage young people in the design and implementation of clinical research about them
Develop a clear, relevant vision and mission	All cases	Cross-cutting all cases
Understand the role of culture in organizational change	Saint Jude Affiliate Program, USA	Choose an affiliate partner that is committed to their community and consistently energetic about engagement
	Youth Trial Boards (United Kingdom, South Africa, Uganda, Zimbabwe)	Selecting core staff with a passion for working with youth embedded the project with the ethos of youth engagement
		Changing institutional culture is a long process full of mediation, compromise, and sometimes standing your ground
		It is important to manage young people’s expectations about how rapidly culture shifts
Community engagement requires an investment mentality	Saint Jude Affiliate Program, USA	Establishing sites was a 18- to 24-month process
	Youth Trial Boards (United Kingdom, South Africa, Uganda, Zimbabwe)	The YTB is an iterative model that is still being shaped
Develop and apply a structured approach	Saint Jude Affiliate Program, USA	Formative assessments are coupled with ongoing site reviews
	Legally-authorized representatives, West Africa	The LAR document was developed using a Delphi consultation process
	Youth Trial Boards (United Kingdom, South Africa, Uganda, Zimbabwe)	A literature review and formative research with young people informed development of the preliminary model
Deploy strong operational and project management	All cases	Cross-cutting all cases
Identify and deploy appropriate resources	All cases	Staff/Administration Champions/owners within sponsoring organization Appropriate timelines
	Saint Jude Affiliate Program, USA	Educational opportunities are available for community partners
	Youth Trial Boards (United Kingdom, South Africa, Uganda, Zimbabwe)	Each project site has a multidisciplinary core team with staff who have prior experience working with youth
		Members of the core team are trained to engage with clinical trials
Leverage community resources	Saint Jude Affiliate Program, USA	Community resources include patient navigators, a family advisory committee, and digital health tools
	Youth Trial Boards (United Kingdom, South Africa, Uganda, Zimbabwe)	Program resources include a referral system for other services to address overall well-being
Communication must be authentic, transparent, and ongoing	Saint Jude Affiliate Program, USA	Information is exchanged on a variety of schedules using a variety of methods about levels of engagement, processes, and outcomes
	Legally-authorized representatives, West Africa	The project will solicit public comment via an online survey that will be open to the global public for 6 weeks
	Youth Trial Boards (United Kingdom, South Africa, Uganda, Zimbabwe)	YTB team members provided feedback on patient information sheets which resulted in development of youth-led information materials
Community engagement is built upon trust	Saint Jude Affiliate Program, USA	Relationships were built through mutual professional interests leveraging existing systems at the time
	L Legally-authorized representatives, West Africa	Recruitment of project participants was achieved through existing networks (bioethicists, pediatric health researchers, and pediatric health policy makers)
	Youth Trial Boards (United Kingdom, South Africa, Uganda, Zimbabwe)	Trust and respect must be established before young people can truly influence and effect change
Success builds on success	Saint Jude Affiliate Program, USA	Be guided by well-defined success that is communicated effectively
		Manage a portfolio that provides payback to all stakeholders
Build the case for novel approaches	Youth Trial Boards (United Kingdom, South Africa, Uganda, Zimbabwe)	It took time to secure the industry funding for the project
Plan sustainability	Saint Jude Affiliate Program, USA	Identify outcomes that are most important to stakeholders
		Maintain buy-in through self-directed improvement and ownership of projects by stakeholders
	Legally-authorized representatives, West Africa	Develop a targeted intervention with an exit strategy
	Youth Trial Boards (United Kingdom, South Africa, Uganda, Zimbabwe)	The project is developing quality standards on youth engagement that will allow assessment of the impact of YTBs
Develop global strategies that work locally	Legally-authorized representatives, West Africa	The project is developing a regional (West Africa) LAR document that allows country-level adaptation
	Youth Trial Boards (United Kingdom, South Africa, Uganda, Zimbabwe)	This was a multi-national project that required developing practice agreements with partners that complied with country-specific regulations and laws

What do the case studies tell us? The descriptions in the case studies support the application of principles governing stakeholder engagement to promote equity in research participation. All groups stressed the importance of coalescing around a shared need and a common vision and mission for addressing that need. The groups also identified several characteristics that are well-documented features of successful project management or change management activities, including deploying appropriate resources, identifying relevant and measurable outcomes, and effective communication. Only one of the three case studies explicitly addresses the role of cultural change in community engagement. Unlike other elements of organizational change, there is no consensus on how to define organizational cultures and limited data on strategies for effecting cultural change [55]. The groups also emphasized the need to invest not only financial resources but also time to establish relationships and build trust with the community. This aligns with other reports [56]. The case studies illustrate that the communities themselves contribute resources to these projects, including resources to address community-specific needs such as pastoral care.

Some elements of the projects are specific to equity in access to pediatric drug development, such as building networks of pediatric specialists or the development of legally-authorized representative guidance documents for minors. However, many of the techniques (e.g., leveraging existing professional relationships, population-specific engagement with research) are applicable to other clinical research engagement beyond pediatric drug research. The approaches highlighted by the case studies are applicable to all pediatric drug development because of the importance of investigator–participant relationships that respects each group’s needs and priorities. Furthermore, sharing the lived experience of study participants adds value to the operationalization of research. These approaches are particularly important when dealing with dispersed, rare, or stigmatized groups because investigators are less likely to be aware of these groups’ needs, preferences, and priorities. The case studies describe specific strategies to address community-level access issues. These included strategies to mitigate geographic barriers (e.g., use of remote access) and to address family and individual needs (e.g., family advisors and pastoral care), and legal status (e.g., in non-parental consent for children in non-family custodial care). Two of the case studies also describe processes for establishing multi-national community engagement activities.

The case studies illustrate how to overcome the multifaceted and systemic barriers to equitable access to research. While egregious breaches of trust have taken place, or continue, in specific settings we need to look beyond them using the approaches demonstrated in the case studies. Addressing “consent bias” among potential participants is not sufficient. Bias among investigators can also be important [57]. Events such as the Tuskegee experience [14], racist medicine in Cape Colony (now South Africa) [58], or contemporary practice in Canada [59] may be used to explain non-participation. However, the focus on consent bias may distract from ongoing unethical practices that need to be addressed using the techniques described in the case studies [10].

The limitations of this paper include the small number of case studies that were selected. The case studies were purposively selected to address different difficulties providing equitable access to research rather than being a systematic survey. Nevertheless, the case studies present strategies for successful engagement. The case studies do not address the amount of resources needed or the methods for procuring these resources.

In summary, the case studies illustrate that all engagement needs appropriate planning and resources. Effective engagement does not happen by accident. Partnerships may be established through existing affiliations (e.g., tertiary hospital centers and community hospitals) or new alliances (e.g., the Youth Trials Boards approaching pharma companies). Our observation also is that all three projects are built upon the strengths of the organization or individuals that developed the project as well as the communities themselves, specifically understanding the network needed for oncology care and research; expertise in law and ethics regarding minor rights and non-parental consent; and the dynamism and enthusiasm of youth participants. The case studies also addressed multiple community-specific challenges including travel and family responsibilities, cultural and linguistic differences, and remote access requirements. The programs have developed strategies for sustainability, including procuring ongoing funding, identifying outcomes important to stakeholders, and employing effective communication strategies to maintain stakeholder buy-in.

## Conclusions

Providing equitable access to pediatric drug developing requires considerable effort. More fundamentally, it starts with an ethos that providing equitable access is not only right but possible. This ethos is reflected in each of the case studies presented in this article. Achieving the possible requires more than a shared belief, it also requires sustained resources, time, and selecting outcomes for success that are meaningful to all stakeholders involved in the project. Equally important, it is essential to communicate successes effectively. When all of these components are brought together, equity can indeed be translated from aspiration into operation.
